# Basophil Activation Test: Old and New Applications in Allergy

**DOI:** 10.1007/s11882-018-0831-5

**Published:** 2018-11-15

**Authors:** Oliver Hemmings, Matthew Kwok, Richard McKendry, Alexandra F. Santos

**Affiliations:** 1 Department of Women and Children’s Health (Paediatric Allergy), School of Life Course Sciences, Faculty of Life Sciences and Medicine, King’s College London, London, UK; 2 Peter Gorer Department of Immunobiology, School of Immunology and Microbial Sciences, Faculty of Life Sciences and Medicine, King’s College London, London, UK; 3 MRC and Asthma UK Centre in Allergic Mechanisms of Asthma, London, UK; 4 Children’s Allergies Department, Guy’s and St. Thomas’ NHS Foundation Trust, St. Thomas’ Hospital, Westminster Bridge Road, London, UK

**Keywords:** Basophil activation test, Diagnosis, IgE, IgG, Allergy, Immunotherapy

## Abstract

**Purpose of Review:**

The basophil activation test (BAT) using flow cytometry has supplanted traditional methods of measuring basophil degranulation using histamine and other mediator release, and can be used for clinical applications as well as to explore the immune mechanisms of effector cell response to allergen. This review discusses the advancements made in clinical, diagnostic and laboratory research of allergy utilizing an ever-evolving BAT.

**Recent Findings:**

Being an in vitro surrogate of the allergic reaction that happens in vivo in the sick patient, the BAT can be used to support the diagnosis of various allergic conditions, such as food, drug, respiratory and insect venom allergies, and the assessment of clinical response to allergen-specific immunotherapy and other immunomodulatory treatments. The BAT can also be used for research purposes to explore the mechanisms of allergy and tolerance at the level of the basophil, for instance by manipulating IgE and IgG and their receptors and by studying intracellular signalling cascade in response to allergen.

**Summary:**

This review covers the applications of the BAT to the clinical management of allergic patients and the increased understanding of the mechanisms of immune response to allergens as well as technological advancements made in recent years.

## Introduction

In the past, testing the basophil response to allergen was focused on the measurement of mediators released by cells into the supernatant in vitro, including histamine and leukotrienes [1, 2]. Technical and analytical limitations, the relatively large blood volume required and low sensitivity [1, 3], together with the discovery and widespread use of flow cytometry techniques have opened scope for new basophil assays, like the basophil activation test (BAT) [2, 4•, 5]. The BAT is a functional assay that measures IgE function, i.e. its ability to induce the activation of basophils in the presence of allergen. The BAT has the potential to closely replicate in vitro type I hypersensitivity reactions, which develop in vivo when allergic individuals are exposed to the allergen, and thus can have clinical applications in the diagnosis and prognosis of allergic disease, alongside research applications. In this review, we will cover the main principle technical aspects related to the performance of the BAT, its clinical applications across different allergic conditions and the use of BAT to explore the immune mechanisms of allergy and tolerance, in order to improve our understanding of the development of allergic conditions and potentially find new targets for treatment and prevention.

## The Basophil Activation Test: Principles and Technical Aspects

The BAT uses flow cytometry to measure the expression of activation markers on the surface of basophils that are upregulated following the cross-linking of IgE antibodies bound to the high-affinity IgE receptor (FcεRI) that result from allergen or anti-IgE stimulation. There are a number of different markers which can be used to identify basophils and to quantify their activation by flow cytometry (Table 1). In addition to the expression of cell surface markers, basophil activation can also be studied by looking at the phosphorylation of certain intracellular molecules, such as p38 mitogen-activated protein kinase (MAPK) or signal transducer and activator of transcription (STAT) 5, which are part of the signalling cascade downstream of IgE and its high-affinity receptor [21, 22]. Other methods of assessing basophil activation, using technologies such as CYTOF [23•] and fluorescent-avidin [24], have been suggested.

**Table 1:** Basophil identification and activation markers

	Marker	Cell expression	Description and gating strategy	Reference
Identification markers	CCR3	Basophils, mast cells, Th2 lymphocytes	Stable marker for identification, may need further differentiation from other cells such as use of CD3	[6–9]
	CD203c	Basophils, mast cells, CD34^+^ progenitor cells	Widely used identification marker and expressed in low levels of resting cells. Upregulation has been reported to be representative of piecemeal degranulation	[10, 11]
	CD123	Basophils, plasmacytoid dendritic cells	Highly expressed in these cells; HLA-DR can be used to differentiate between HLA-DR^+^ dendritic cells	[12, 13]
	IgE	Basophils, monocytes, dendritic cells	Other cells expressing high affinity IgE receptor FcɛRI can be differentiated by also using HLA-DR. High variability in individuals has been reported	[6, 14]
	CRTH2	Basophils, eosinophils, Th2 lymphocytes	Differentiation from eosinophils by side scatter or T lymphocytes by CD3 is reported	[15]
Activation markers	CD63	Basophils, mast cells, platelets, macrophages	Widely used activation marker and an accurate marker of anaphylactic degranulation	[7, 11, 16, 17••]
	CD107a, CD107b	Various cell types including basophils, mast cells, T cells and NK cells	CD107a and CD107b was found to be only expressed by activated basophils and its upregulation was similar to CD63. CD13 and CD164 has an expression profile comparable to CD203c	[18]
	CD13	Basophils, myeloid cells		
	CD164	Basophils, CD34^+^ progenitor cells		
	CD69	Basophils, lymphocytes, neutrophils, monocytes, eosinophils	Expressed progressively when stimulated with IL-3; however, found to be weakly expressed to IgE mediated stimulation	[19, 20]
	p38 MAPK, STAT5	Various cell types	Basophilic phosphorylation of intracellular molecules can alternatively be used to measure basophil activation	[21, 22]

The BAT can be performed using whole blood or isolated peripheral blood mononuclear cells (PBMCs), which include the basophils. PBMC isolation can be performed using density gradient separation and additional negative selection using magnetic particles allows enrichment for basophils [25]; however, there is an increased risk of cell loss from the centrifugation steps and excessive handling of the cells may cause background activation [26]. Flow cytometry and fluorescent staining techniques have allowed for the specific investigation of basophils in whole blood without the need for further manipulation [3]. Additionally, the use of whole blood as opposed to isolated basophils may be more physiological and more closely resemble the in vivo environment of blood basophils, with other factors present, such as blocking antibodies, that may play a role in the allergic or non-allergic phenotype of individuals [26].

Whole blood BAT should ideally be performed within 4 h of blood collection to maximize viability and functionality of basophils [27, 28], as basophil reactivity decreases considerably over time [29]. If a longer time is needed between blood collection and the performance of the BAT (e.g. multicenter studies or clinical centers located far from reference laboratory), blood can be processed within 24 h [30]. Mukai and colleagues [23•] have shown that BAT performed on blood stored in heparin at 4 °C at 4 h and at 24 h following blood collection did not show significant differences and that transportation during this period of time did not significantly affect the results. Collection of whole blood for BAT is usually done in heparin; other anticoagulants such as ethylenediaminetetraacetic acid (EDTA) or acid citrate dextrose (ACD) can prevent basophil degranulation [4•]. EDTA, for example, can chelate calcium, which can be reversed by the addition of extracellular calcium in later incubation steps; however, in possibly different concentrations than the physiological concentration in the original blood sample. Priming of basophils with IL-3 can help to enhance IgE-mediated CD63 responses to allergen but can induce non-specific upregulation of CD203c and p38 MAPK signalling pathways [21, 28, 31]. Sturm and colleagues also observed that pre-warming samples and reagents at 37 °C for 10 min before performing BAT to enhance basophil responses had no effect [28].

Allergen source is another critical factor in both clinical and research applications of the BAT. Allergen stimulants range from crude extracts to recombinant or purified single allergen sources. Standardized preparations are recommended when comparing performance data from different laboratories and when performing tests over time [4•]. The availability of recombinant allergens for testing in the BAT may be limited but they have the greatest stability and consistency compared to crude allergen or extracts and can help improve diagnostic accuracy in some cases [14, 32]. To test for drug allergy, pure drug preparation used for parenteral administration is preferred and dilutions should be prepared shortly before performing the BAT [4•].

Testing a dose-response consisting of at least five different allergen concentrations, for instance in 10-fold increments, is recommended as opposed to a single allergen concentration given the variability of basophil responses between individuals. This variability is partly due to the complexity of antigens, varying affinity of IgE for allergen epitopes and intrinsic basophil sensitivity [2, 33]. Consequently, the dose-response profile can vary markedly from a typical bell-shaped dose-response curve [2]. The results of the BAT from a dose-response curve can be expressed as basophil reactivity, basophil sensitivity or both [33]. Basophil reactivity can be measured using %CD63+ basophils at a given concentration or using CD-max, i.e. the concentration at which maximal basophil activation occurs. Conversely, basophil sensitivity can be expressed as either EC50 (the concentration at which 50% of maximal basophil response occurs) or CD-sens (defined as the inverse of EC50 multiplied by 100 and this can be calculated from the slope of the dose-response curve) [34]. The area under the dose-response curve has been used more recently to assess basophil reactivity and sensitivity simultaneously [35].

About 5–10% of individuals have non-responding basophils, in which no upregulation of CD203c or CD63 occurs in response to IgE-mediated allergen stimulation, but only to non-IgE-mediated stimulants [26]. It is therefore recommended to include both IgE-dependent (e.g. anti-IgE or anti-FceRI) and IgE-independent (e.g. fMLP or ionomycin) positive controls in the BAT [36]. Furthermore, a negative control consisting of stimulation buffer alone should also be included to assess the level of background or spontaneous activation of basophils [26]. If IL-3 is used, an additional control for IL-3 in the stimulation buffer should be included.

The transition of the BAT from the research laboratory to clinical practice is dependent on the standardization and quality assurance of the laboratory procedure, flow cytometry and data analyses, in addition to the clinical validation of the diagnostic performance of BAT to different allergens [4•, 14]. Automation of flow cytometry data analyses can help improve the efficiency and transparency of analyses and the reproducibility of the data [37••]. Bioinformatics tools and resources including raw data are increasingly becoming available to the public, which can further facilitate standardization and sharing of methodology [14]. Currently, an international task-force from the EAACI has gathered experts with extensive experience in the clinical application of the BAT to start addressing the quality assurance of the BAT. The assessment of the cost-effectiveness and impact on clinical-decision making is likely to be central to the regulatory approval of the BAT for clinical use and deserves further research [33].

## Clinical Applications of the Basophil Activation Test

The clinical history is the key factor to accurately diagnose allergy. With a likely culprit allergen and an immunologic mechanism in mind, the documentation of the presence of IgE or a cell-mediated reaction using serum-specific IgE (sIgE) and/or skin testing supports the diagnosis [38–42]. Despite being well-established, skin testing and sIgE have practical limitations—for instance, skin testing requires intact skin and anti-histamine cessation, and both skin testing and sIgE detect sensitization, which does not equate to clinical allergy [43]. If the diagnosis is equivocal following the clinical history and skin and/or sIgE testing, a provocation test or challenge needs to be performed under clinical supervision (e.g. oral food challenge in food allergy, drug provocation test in drug allergy). The challenge is labour and resource-intensive and often stressful for the patient as it has the risk of inducing allergic reactions, including cutaneous, oral, gastrointestinal, respiratory and cardiovascular symptoms [44••, 45••, 46••]. Across all types of allergic disease, an in vitro assay that could represent the allergic reaction in vivo better than skin testing and sIgE would be most useful. In IgE-mediated allergy, the BAT emerges as such as it can potentially reproduce the immediate-type allergic reaction in the test tube.

### BAT in the Diagnosis of Food Allergy

Skin prick test (SPT) and sIgE have high sensitivity but low specificity to diagnose food allergy. Although 95% positive predictive value (PPV) cutoffs have been determined for certain allergens and certain populations with enhanced specificity, the majority of patients assessed for food allergy have results below such cutoffs, and even when using specific IgE to allergen components, the results can be equivocal. The BAT has shown to be more accurate than IgE sensitization tests and able to distinguish individuals that were clinically allergic from those who were tolerant albeit sensitized in various studies, with high specificity ranging between 75 and 100% and high sensitivity ranging between 77 and 98% [45••, 47, 48]. In a large peanut allergy study, BAT was externally validated in a new independent population and showed 100% specificity [45••]. This high specificity means that a positive BAT to peanut confirmed peanut allergy and dispensed oral food challenge (OFC). Thus the reduction in OFC was mainly a reduction in positive OFC, sparing patients from experiencing allergic reactions [45••]. Recent studies have shown that BAT has high specificity in diagnosing allergy to tree nuts, higher than SPT and sIgE to individual allergens for instance in the case of hazelnut allergy [49]. BAT to single allergens can enhance the diagnostic performance of BAT for some food allergies, such as Ara h 2 and Ara h 6 to diagnose peanut allergy [50], with the caveat that some allergic patients may not be sensitized to the allergens being used as stimulants in the BAT, potentially leading to false negative results in these selected cases.

BAT also has utility that goes further beyond diagnosing and monitoring food allergy. It can also relate to the severity of allergic reactions in that patients with more severe reactions show a greater proportion of activated basophils and patients reacting to trace amounts of the allergen show a greater basophil sensitivity, i.e. their basophils start reacting at lower allergen concentrations [51, 52, 53].

### BAT in the Diagnosis of Drug Allergy

The diagnosis of drug allergy poses additional challenges compared with the diagnosis of allergy to large protein allergens, such as food and airborne allergens. The clinical phenotype of allergic reactions to drugs is more diverse and the underlying mechanisms can fall into types of hypersensitivity beyond type I hypersensitivity. However, diagnostic difficulties specific to drug allergy also pose additional opportunities for improved methods for testing such as the BAT. For instance, skin testing to drugs, particularly intradermal testing, incurs a significant risk of systemic reactions, including anaphylaxis. Furthermore, sIgE testing is not possible to both the native drug and all its metabolites, many of which maybe the culprits for the allergic reaction [54]. Drug provocation tests (DPTs) to certain drugs are impractical or unethical particularly in the context of anaphylaxis under general anaesthesia. Therefore, in these instances, BAT may be the only diagnostic tool available and is often a cheaper and safer alternative to other tests [55, 56].

Several studies over the last 15 years have reported the diagnostic accuracy of BAT for allergy to a range of drugs including betalactams [36, 57, 58], quinolones [59–61] and neuromuscular blocking agents (NMBAs) [62, 63] (Table 2). More recently, BAT has shown to be useful in the diagnosis of omeprazole allergy: skin tests alone showed sensitivity of 66.7% and specificity of 100%, while the addition of the BAT increased the sensitivity to 73.8% without a reduction in specificity [71]. BAT to clavulanic acid showed high positive predictive value (PPV) in a recent study and thus added value to BAT to amoxicillin when managing patients with suspected amoxicillin-clavulanic acid allergy [72•]. It has been suggested that the BAT may be able to identify the component of a vaccine responsible for immune-mediated adverse reactions [73] and to confirm whether an adverse reaction following transfusion of blood components is immune-mediated [74–76].

**Table 2:** Diagnostic performance of the basophil activation test on different allergic conditions

Allergen		Number of participants	Cutoff values	Sensitivity (%)	Specificity (%)	Publication
Food	Peanut	104	≥ 4.78% CD63+	97.6	96	[45••]
	Cow’s milk	50	CD203c SI ≥ 1.9	89	83	[64]
	Egg	67	≥ 5% CD63+	77	100	[48]
Drug	BetaLactams (various)	24	≥ 5% CD63+	55	80	[36]
	NMBA (Rocuronium)	59	≥ 4% CD63+	80	96	[65]
	Quinolones (ciprofloxacin, moxifloxacin and levofloxacin)	63	≥ 5% CD63+ CD203c SI ≥ 2	71.1	88	[66]
Venom	Wasp	34	Determined but not reported	85.3	83.3	[67]
	Bee	23	Determined but not reported	91.3	90.0	[67]
Airborne	Birch pollen	62	Response to AIT assessed using the BAT with results expressed as basophil allergen threshold sensitivity			[68]
	Timothy grass pollen	24	Response to AIT assessed using the BAT with results expressed as basophil allergen threshold sensitivity			[69]
	*D. pteronyssinus*	13	Not determined	85	93	[70•]

The application of BAT in drug allergy goes further than standard diagnosis to possibly serving as a biomarker for anaphylaxis following drug desensitization. Drug desensitization is imperative for allergic patients requiring full therapeutic doses of lifesaving medication [77] and follows a step-wise protocol administering incremental doses of the drug [77]. For instance, BAT has been used to successfully identify patients allergic to platinum compounds with high risk of adverse reactions during drug desensitization with increased CD203c expression being indicative [78].

### BAT in the Diagnosis of Chronic Urticaria

Chronic urticaria (CU) is largely idiopathic and often spontaneous and exhibits heterogeneity in induction, duration and mechanisms. A subset of CU patients have an autoimmune pathophysiology due to the presence of autoantibodies towards IgE or its high affinity receptor FcεRI [79]. Moreover, the presence of anti-DsDNA antibodies, IgE and IgG targetted towards thyroid peroxidase have been identified in the sera of CU patients [80, 81]. The confirmation of the autoimmune nature of CU demands a functional assay for diagnosis, for which the autologous serum skin test (ASST) has been relied upon. However, this is an in vivo test with a risk of accidental infection and whom test results do not always correlate with other in vitro assays [82, 83]. Therefore, the BAT has been suggested as an in vitro surrogate for ASST, to diagnose and monitor patients with suspected CU. In previous studies, both CD63 and CD203c expression on the surface of basophils was increased following stimulation with sera from CU patients and BAT showed to be a functional test for the detection of active autoantibodies [84, 85]. A recent study has shown that both ASST+/BAT+ urticaria patients often showed the most active disease state, in line with urticaria activity score (UAS). Thirty-two percent of ASST+/BAT+ patients, as opposed to 16% ASST+/BAT− patients required higher dose of antihistamines joint with third-line treatment (cyclosporine A or omalizumab) [86].

### BAT in the Diagnosis of Venom Allergy

BAT reports both high sensitivity (85–100%) and specificity (83–100%) to diagnose hymenoptera venom allergy [67, 87, 88]; however, testing for sIgE to the major allergens of bee and wasp venoms Api m 1 and Ves v 5, respectively, reduced the impact of BAT in a large number of suspected cases. Still, BAT has found greatest success in venom allergy when solving unique diagnostic issues. For instance, a small proportion of patients with a clinical history of venom allergy report undetectable sIgE and negative skin tests. However, current guidelines do not advise on how to pursue diagnosis in these patients, further complicated by the unethical nature of sting provocation tests under these circumstances. BAT has proven effective in diagnosing approximately 80% of these patients, a marked improvement when diagnosing venom allergy with skin tests alone [89].

Another nuance of the diagnosis of hymenoptera venom allergy is the double positivity to both wasp and bee venom, where up to 60% of patients exhibit sIgE to both. In order to progress to venom immunotherapy (VIT), determination of the responsible allergen is imperative. The BAT consistently demonstrates lowest levels of double positivity when compared to other diagnostic methods and where double positivity is apparent, BAT is often able to identify the dominant allergen [90, 91]. Interestingly, sIgE determination to Api m 1 and Ves v 5 reduces true double sensitization to 50% of cases of double positivity, but BAT still appears to add extra information [92•]. This suggests that assessing basophil reactivity utilizing these recombinant allergens as stimuli may be key in determining the culprit allergen source.

### BAT in the Diagnosis of Respiratory Allergy

Allergic reactions to inhaled allergens are heterogeneous and can be complex due to the diversity of potential allergens that patients are naturally exposed to and the number of tissues that can be affected in a localized manner. sIgE quantification and SPT have long been considered sufficient to support the diagnosis of respiratory allergies; however, patients who suffer from local allergic rhinitis can have undetectable levels of sIgE and negative skin tests, making it hard to differentiate between allergic and non-allergic rhinitis. In these patients, BAT has proved more sensitive and able to diagnose IgE-mediated allergy despite the apparent absence of allergen-sIgE systemically [70•]. Moreover, BAT has been used to explore unique aspects of allergic rhinitis and allergic asthma with basophil sensitivity (as expressed by CD-sens or EC50) showing concordance with nasal provocation titre [93] and bronchial provocation threshold respectively, confirming the clinical relevance of the allergen in driving the respiratory symptoms [94].

### BAT in the Follow-up of Patients Submitted to Allergen-Specific Immunotherapy and Other Immunomodulatory Treatments

Determining basophil response to allergens is a powerful tool when monitoring the effects of allergen-specific immunotherapy (AIT) and also of other immunomodulatory treatments, such as anti-IgE [93, 95, 96]. Basophil sensitivity (as measured by CS-sens) has shown to be reduced following AIT to aeroallergens such as birch pollen [97, 98] and timothy grass pollen [99], and following AIT to insect venom [100, 101]. A drop in basophil sensitivity has also been reported following treatment with omalizumab across a range of allergies including allergy to peanut, cat and *Aspergillus* [102–104]. Interestingly, individuals with a higher antibody-specific activity (higher percentage of allergen-specific IgE), displayed a greater efficacy to anti-IgE treatment, as determined by BAT [103]. This association is rational, as a higher percentage of allergen-specific IgE means it is more readily decreased following omalizumab administration. However, the intrinsic sensitivity of the basophil itself also appears to be modified during anti-IgE therapy, and competes with antibody specific activity, to alter the basophils response to challenge. The intrinsic sensitivity of a basophil, can be correlated with the expression of basophil spleen tyrosine kinase (Syk), an enzyme essential for IgE-mediated histamine release, and appears to increase during omalizumab treatment [105••]. This is counterintuitive to the overall decrease in basophil (allergen threshold) sensitivity as previously described in response to omalizumab, but means Syk expression could be a potential biomarker for predicting the clinical efficacy of omalizumab [106••].

## Using the Basophil Activation Test as a Tool to Explore the Immune Mechanisms of Allergic Disease

**Fig. 1:**
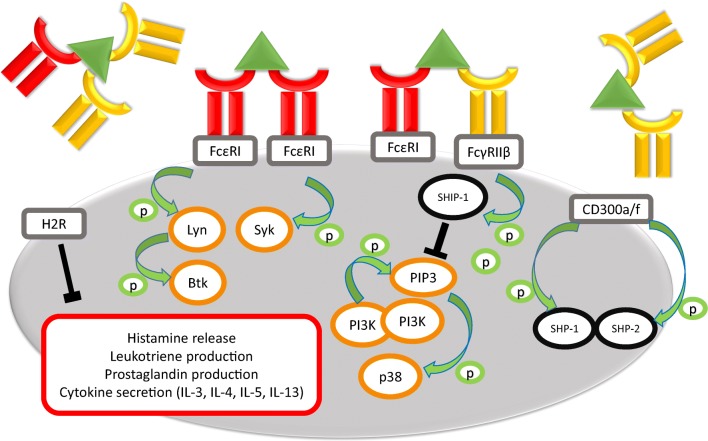
Mechanisms of basophil response to allergens. A schematic diagram of basophil activation/inhibition in response to antibody crosslinking with allergen. IgE (red) and/or IgG (yellow) ligate cell surface receptors (grey boxes) and bind allergen (green triangles), inducing phosphorylation (green arrows/circles) of activatory (orange circles) or inhibitory (black circles) signalling proteins. Activation of basophils induces the secretion of potent cell mediators (red box), unless inhibited by intracellular processes (black “T”)

The BAT transitions between the clinic and the laboratory in part due to its preservation of complex patient phenotypes, but also to the diverse immunological targets which can be assessed using this assay (Fig. 1). IgE-mediated basophil activation follows sensitization in which IgE binds to FcεRI with high affinity and occurs when stimulation allergen or anti-IgE antibodies results in cross-linking of IgE molecules, phosphorylation of immunoreceptor tyrosine-based activation motif (ITAMs) in the intracellular portion of the beta and gamma chains of FcεRI and subsequent cascade of phosphorylation of components of the intracellular activatory signalling pathway. Parallel inhibitory components can also be phosphorylated depending on concomitant co-stimulation, namely of immunoreceptor tyrosine-based inhibition motif (ITIM)-associated receptors on the surface of basophils and may have an important role in basophil regulation. One of the inhibitory receptors of interest in antibody-mediated responses is CD32B, which can bind IgG and potentially allow cross-linking between IgE and IgG and suppress IgE-mediated basophil activation. IgG and other antibody isotypes other than IgE can interfere with allergen-IgE interaction also by blocking and competing for binding to the allergen in the extracellular space. [107, 108]

The key to the success of BAT in understanding the mechanisms of allergic disease is the ability to characterize the surface proteins expressed by basophils and how these modulate the response to allergen stimulation or challenge. The BAT allows for FcεRI expression to be quantified [109, 110] as well as the expression of other Fcgamma receptors (CD16, CD32, CD64) in the steady state and also in response to activation or challenge [111, 112]. The inhibitory potential of CD32 isoforms has been investigated using the BAT, revealing a complex system in which combinations of IgG isotypes and IgG concentrations elicit different levels of histamine release following blocking of CD32A or CD32B. Expression of Fcgamma receptors on basophils has been investigated in the context of allergy, for instance Meknache et al. [113] observing a lower expression of FcγRIIIB on basophils from atopic dermatitis patients compared to patients with allergic rhinitis, asthma or chronic urticaria. The importance of Fcgamma receptor expression may have wider implications, as basophils from myelogenous leukaemia patients have an aberrant expression of CD64 [114]. The functional implications of these studies have yet to be fully elucidated.

Similarly, it is possible to measure receptor-bound antibodies on the surface of effector cells [115]. A detailed study by Eggel et al. [116] investigated the expression of surface-bound IgE in response to omalizumab treatment. The loss of surface-bound IgE mediated by omalizumab treatment resulted in ablated basophil activation, accompanied by the downregulation of FcεRIα from the surface of basophils and a reduction in basophil numbers. The re-sensitization of basophils was performed by stripping surface IgE followed by addition of allergen-specific IgE before allergen challenge. The re-introduction of IgE induced the activation of basophils, further emphasizing the key mechanism of IgE-FcεRI-mediated allergy. Furthermore, the authors expanded this model to test the effectiveness of other IgE inhibitors, utilizing the BAT to compare therapeutics under identical experimental conditions.

Basophil response to allergen stimulation can be modulated by the characteristics of allergen-specific IgE that is bound to its receptors, namely its concentration, specificity, clonality and affinity for allergen and this immunomodulation can be studied in vitro [117, 118]. Christensen et al. generated 31 Der p 2-specific recombinant IgEs and showed that specificity and specific activity influence basophil reactivity whereas affinity and clonality influence basophil sensitivity [119•]. In order to improve our understanding of basophil response to allergen, using single allergens or epitope-containing peptides can add valuable information to the results obtained with allergen extracts [120]. Hayen et al. compared basophil responses to recombinant isoforms of Ara h2, Ara h 6, Ara h 7 and crude peanut extract, implicating Ara h 7.0201 as a dominant epitope for peanut allergy [121]. This work was built upon previous work on peanut allergy [118, 120] and additional studies have been performed investigating other foods [122, 123]. Greater understanding of peptides and epitopes which elicit allergic responses are key for both improved diagnostic tests and potential novel treatments.

Basophil activation can occur in an IgE-independent manner and the BAT can be performed to confirm the mechanism of activation to allergen. For instance, Aranda et al. tested whether basophil activation to quinolones resulted from an IgE-mediated mechanism using the PI3K inhibitor wortmannin [66]. The addition of wortmannin to the BAT experiments inhibited basophil activation to ciprofloxacin, moxifloxacin, levofloxacin and anti-IgE treatment, but inhibition was not observed with fMLP treatment [66], confirming the IgE-mediated nature of the reactions to the quinolones.

The BAT can also be used to explore the mechanisms of AIT at the basophil level. Reduction in basophil reactivity and in basophil sensitivity have both been reported with this treatment in patients with allergy to respiratory, food and venom allergens [99]. Remarkably, during oral peanut immunotherapy, the reduction in basophil reactivity was observed not only to the culprit allergen but also to a bystander allergen (e.g. egg) and IgE-mediated but not non-IgE mediated controls, suggesting basophil anergy [124•]. A study of allergic rhinitis patients sensitized to house dust mite (HDM) and mugwort showed that while desensitization was achieved to mugwort, no change was seen in reactivity to HDM after 24 months of AIT [125]. Discrepancies between study results may be in part due to natural allergen exposure (daily ingestion versus seasonal exposure), nature of the allergen molecules and route of administration [126], with oral IT to peanut performed by Thyagarajan et al. [124•] and subcutaneous IT performed by Kim et al. [126].

A number of studies have directly linked AIT to changes in immunoglobulin levels that in turn reduce basophil sensitivity. In particular, the significant increase in serum IgG in response to immunotherapy reduces basophil sensitivity, probably through the competition for allergen binding [98, 127, 128]. IgG4 in particular is regarded as possibly having an ‘immunoregulatory’ role, inhibiting allergic responses and maintaining immunological tolerance. Using a passive sensitization BAT in which IgE stripping from the receptors on the surface of basophils is followed by addition of patients’ serum, Santos et al. [129] revealed the inhibitory role of allergen-specific IgG4 in peanut-sensitized but tolerant individuals and peanut-allergic individuals treated with oral peanut immunotherapy. Chan et al. [130] identified IgG antibodies specific for soluble IgE in serum and IgE bound to FcεRI in non-allergic asthmatic patients. IgE binding of allergen was inhibited in sera containing IgE-specific IgG molecules, while depletion of IgG from sera ablated the inhibition of basophil activation. Thus, the BAT has elucidated that there are multiple potential mechanisms of basophil inhibition which are mediated by IgG-isotype antibodies.

The relevance of receptors not commonly associated with IgE can also be investigated using the BAT. Ligation of histamine receptors expressed by basophils (H1R, H2R and H3R) have been shown to induce basophil activation [16] which may be relevant in IT directed to insect venom [131]. Suppression of basophils mediated by histamine receptor 2 (HR2) has been proposed as an alternative mechanism of ‘immuno-dampening’ following immunotherapy [131]. Another inhibitory receptor of interest is CD300a (IRp60) which is upregulated in response to IgE/FcεRI cross-linking [10]. TSLP receptor has recently been identified as being upregulated by basophils in allergic rhinitis patients in response to allergen challenge [11], adding to the growing number of receptors implicated in allergic responses beyond FcεRI.

Although IgE:FcεRI-mediated basophil activation has been established, the signalling events leading to CD63 upregulation and basophil degranulation are yet to be fully elucidated. The BAT allows for the phosphorylation of key activatory or inhibitory intracellular signalling proteins to be assessed simultaneously and in relation to CD63 and CD203c upregulation [21]. Verweij et al. [22] implicated STAT5 signalling as an activator of basophils in response to birch pollen (rBet v 1), but intriguingly, this required the presence of IL-3. Christensen et al. [12] investigated the role of signalling proteins in the context of AIT. Desensitized basophils displayed significantly reduced phosphorylated p38MAPK, which in an allergen-specific manner, resulted in reduced activation during allergen challenge. Changes in Syk expression via IgE in response to omazulimab treatment may also contribute to inhibition of allergic responses [13], but further work is required to understand the complex signalling pathways associated with basophil activation and inhibition and their modulation following allergen stimulation.

## Conclusions

The BAT reproduces IgE-mediated allergic reactions in vitro, is a useful system for both clinical and research applications and has surmounted the old mediator release assays. Clinically, it can support the diagnosis of IgE-mediated allergic conditions and monitor patients over time and their response to immunomodulatory treatments. For research, BAT offers endless possibilities of studying the various components of the IgE-mediated allergic reaction and their modification, allowing dissection of the mechanisms of allergy and its suppression to improve our understanding and pave the way for the discovery of new targets for treatment and possibly prevention of allergic disease.
